# The relationship between ageing and changes in the human blood and brain methylomes

**DOI:** 10.1093/nargab/lqac001

**Published:** 2022-02-02

**Authors:** Patrick Bryant, Arne Elofsson

**Affiliations:** Science for Life Laboratory, Tomtebodavägen 23, Box 1031 17121, Solna, Sweden; Department of Biochemistry and Biophysics, Stockholm University, Svante Arrhenius väg 16C SE-106 91, Stockholm, Sweden; Science for Life Laboratory, Tomtebodavägen 23, Box 1031 17121, Solna, Sweden; Department of Biochemistry and Biophysics, Stockholm University, Svante Arrhenius väg 16C SE-106 91, Stockholm, Sweden

## Abstract

Changes in DNA methylation have been found to be strongly correlated with age, enabling the creation of ‘epigenetic clocks’. Previously, studies on the relationship between ageing and DNA methylation have assumed a linear relationship. Here, we show that several markers show a non-linear behaviour. In particular, we observe a tendency for saturation with age, especially in the cerebellum. Further, we show that the relationships between significant methylation changes and ageing are different in different tissues. We suggest a straightforward method of assessing all methylation-age relationships and cluster them according to their relative fold change. Our fold change selection outperforms the most common epigenetic clocks in predicting age for the cerebellum, but not for Blood or the Frontal Cortex. Further, we find that the saturation of methylation observed at older ages for the cerebellum explains why epigenetic clocks consistently underestimate the age there. The findings imply that assuming linear correlations might cause biologically important markers to be missed.

## INTRODUCTION

Ageing is a process experienced by most organisms, but why ageing occurs and how to define it on a biological level remains elusive. Epigenetic changes, such as alterations in DNA methylation, histone modifications and changes in chromatin states, are related to ageing in animal models (1), suggesting an epigenetic role in regulating lifespan (2).

In particular, the role of DNA methylation on CpG sites has been used to relate chronological age (CA) to DNA methylation age (DNAmAge), deemed the ‘epigenetic clock’ (3). The original clock, by Steve Horvath, uses a linear combination of 353 CpG methylation sites selected by elastic net regression (4) and can predict CA with a correlation of 0.96 and an average error of 3.6 years using 51 healthy tissues. Another model by Hannum (5), using only blood, uses the same principle to obtain a correlation of 0.96 and an accuracy of 3.9 years.

Non-linear models have been used to improve the DNAmAge predictions resulting in higher correlations between DNAmAge and CA (6,7). These models were built using preselected methylation markers, using elastic net regression and analysis of Pearson correlations, thereby favouring selecting probes based mainly on only linear relationships between CA and DNA methylation levels.

However, reports of ‘systematic underestimation of the epigenetic clock and age acceleration in older subjects’ exists (8). This could be explained if the relationship between methylation change and CA is non-linear. Although it may have a sizable linear regime since the non-linear effect is most prominent in older individuals. The underestimation is observed in all examined tissues but most apparent in the cerebellum.

Methylation levels at only a few CpG sites, used in epigenetic clocks, can be sufficient for predicting ageing and accompanied death risk to a certain extent (3,5,9,10). Attempts to analyse the real effect on gene expression from these markers experimentally found hypomethylation being associated with ageing (11). Longitudinal studies have further attempted to validate methylation markers by looking at marker consistency across measurements and their relation to ageing and disease (12,13).

The studies mentioned above assume a linear relationship between ageing and important methylation markers by either correcting for ageing or correlating age with methylation change in a linear combination. Even though a linear feature combination allows for a non-linear prediction, selecting features using linear methods favours constant, linear relationships. However, there is no biological reason that this assumption is valid, i.e. that the most important relationships between ageing and methylation are linear. Therefore, we here compare the relationship between methylation markers and ageing without making assumptions on the relationship between methylation and ageing.

## MATERIALS AND METHODS

### Data preparation

**Figure 1.**
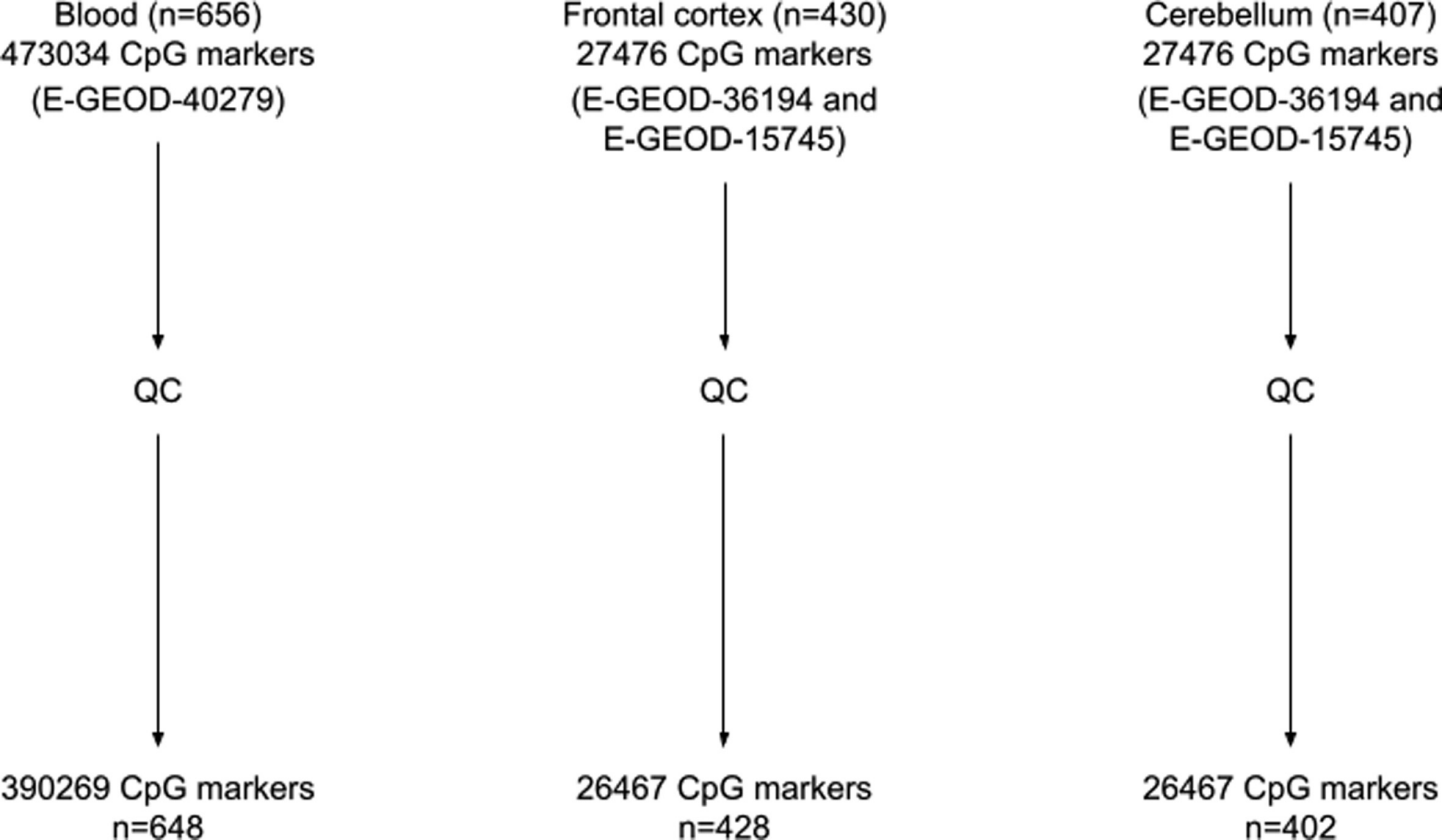
Flow chart of data preparation for blood, frontal cortex and cerebellum, respectively. After quality control (removing outlier samples, cross-reactive probes, probes with known overlapping SNPs and markers with missing values), 648 samples are available for blood, 428 for the frontal cortex and 402 for the cerebellum.

**Figure 2.**
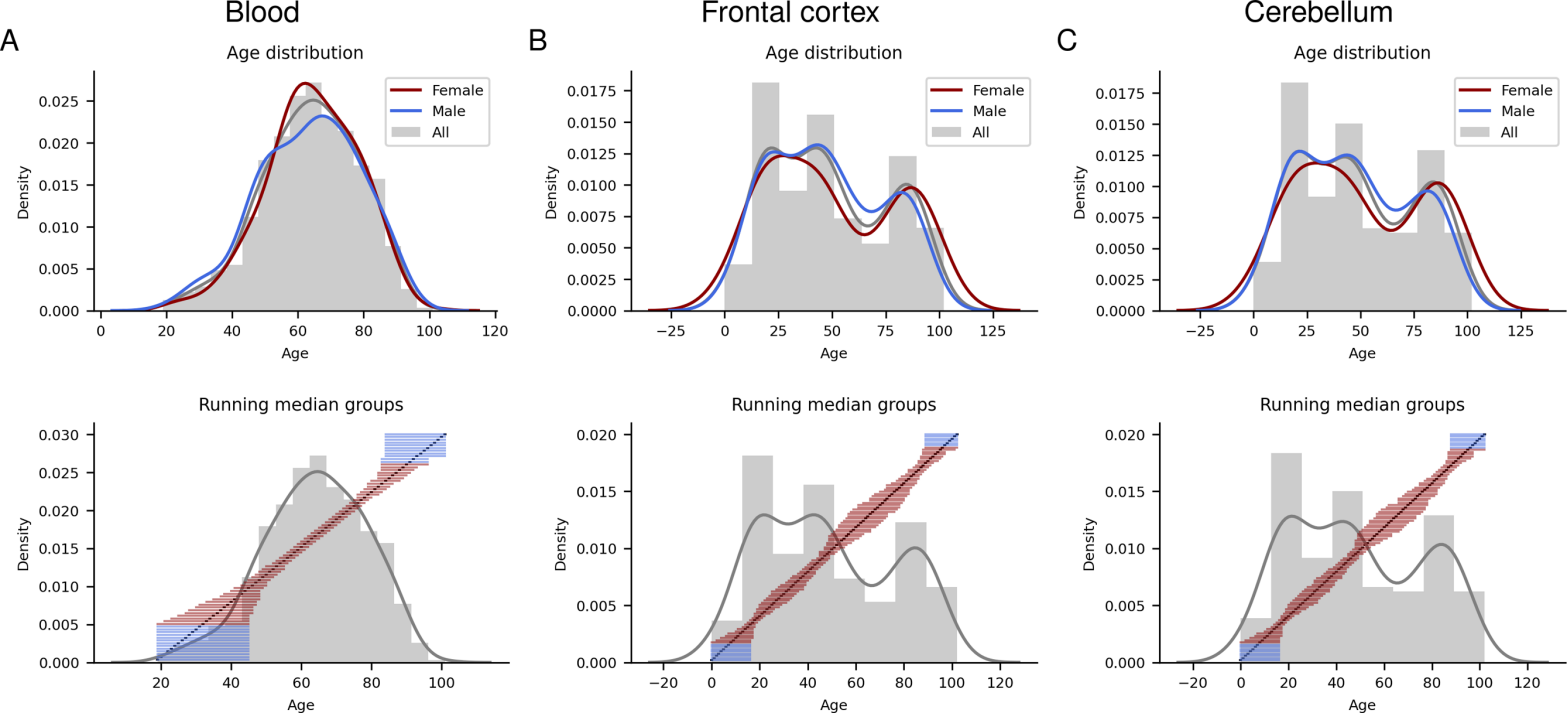
The top row shows the age distribution of all samples and divided by sex for blood (**A**), frontal cortex (**B**) and cerebellum (**C**) respectively. The ages range from 19–101 for blood and 0–102 for the brain samples. The bottom row displays the inclusion limits for the running median groupings using the closest 10% of samples for each year displayed as horizontal blue and red lines for blood (A), frontal cortex (B) and cerebellum (C), respectively. The red lines show the groups used in the analysis, while blue show those that were excluded. The age histogram in grey and the age are grouped marked in black, blood, frontal cortex and cerebellum, respectively.

We utilise Illumina Infinium 450k Human DNA methylation profiles from a set of 656 blood samples from healthy patients from the ArrayExpress database (E-GEOD-40279, (5)) and Illumina 27k Human DNA methylation profiles from 428 frontal cortex and 402 cerebellum samples, both from healthy patients. Both brain regions contain samples from both E-GEOD-36194 and E-GEOD-15745 (14) (Figure 1). The samples span a vast range of ages (19–101 years for blood and 0–102 years for the frontal cortex and cerebellum, Figure 2, and have balanced sex distributions.

In brief, for the blood samples (5), the methylation status of all autosomal chromosomes have been measured using the Illumina Infinium 450k chip. The relative intensity difference between the methylated and unmethylated probe values were adjusted with Illumina’s GenomeStudio for internal controls.

For the E-GEOD-15745 samples, briefly, 150 neurologically normal Caucasian subjects were used to collect tissue samples from the cerebellum and frontal cortex in frozen form. For E-GEOD-36194, frozen tissue samples were obtained from 318 healthy patients from the frontal cortex and cerebellum.The methylation status was interrogated using the illumina 27k methylation array and analysed using Illumina’s GenomeStudio for both of these studies (14).

Since no normalization of the beta values was performed (nor in the original studies from which these originate), the beta values from a study can be used directly without any potential bias that may arise due to different normalization procedures, increasing the robustness of the analysis. Further quality control was performed by removing probes with known overlapping SNPs (17 032 out of 473 034 for blood and 336 out of 27 476 for brain) and cross-reactive probes (15) (28 219 out of 456 002 for blood, 1009 out of 27 476 for blood) and markers with more than ten missing beta values (37 514 out of 456 002 from the blood samples but 0 out of 26 467 from the frontal cortex and cerebellum samples). We then calculated the mutual information (16) for the beta-value distribution of each sample by comparing it with the mean beta value distribution. We removed samples with a mutual information score of less than three standard deviations away from the mean (eight from blood, two from frontal cortex and five from cerebellum, see Supplementary Figure S1).

### Creation of age group running medians

To investigate not only linear relationships between marker methylation change and age. We create a running median of the methylation beta values for each probe. The median is less sensitive to outliers compared to an average. To avoid grouping effects, we select 10% of the closest samples by age for each age with steps of one year from the lowest to the highest age. For example, the starting age for blood, 19, only has one sample, why samples up to the age of 45 are included in the median calculation. By including samples selected by age distribution, it is ensured that each calculation has equal sampling.

To avoid edge effects that may arise due to low sample representations at high and low ages, where the samples are fewer, only the groups whose midpoints are close to the ages being represented were used in the analysis (ages 32–90 years for blood, 8–96 for the frontal cortex and 8–95 for the cerebellum). From these age group medians, which we call the running medians, the maximum fold change (FC) for each marker is assessed, by calculating the relative change between the minimum and maximum methylation medians.

### Statistical analysis of running median age groups

A two-sided t-test was made by comparing the samples used to calculate the maximum FC observed for each marker. The markers with a false discovery rate (FDR) below 0.05 (according to the Benjamini–Hochberg procedure (17) in statsmodels (18)) and having a FC higher than two were selected.

To ensure that the median values are stable, we analysed the standard deviation for each interval used. We compute the relative standard deviation by dividing the standard deviation with the median in each interval. If the average relative standard deviation is >0.5, we consider the spread too large and do not include this methylation marker in the analysis.

### Absolute value and overlap selection

The procedure used for the FC was also used to calculate the maximum change in beta values for each marker. The points resulting in the maximum FC are identical to those resulting in the maximum difference, meaning that the subsequent analysis of FDR and relative standard deviations apply here as well. All maximum beta value changes were calculated, and the markers whose maximal change in running median were at least 0.2 were selected. In addition, the overlapping markers between the FC and absolute value selections were selected to analyze a joint selection process.

### Gradient analysis

The gradients of the running medians were calculated to analyze the tendency for marker beta values being linearly related to ageing. The beta values for the gradients were normalised with the highest beta value for each marker, to obtain the same relative change for each marker and thus make them comparable. The gradients were thereafter clustered using *k*-means clustering (19). To select the number of clusters, t-SNE embeddings were computed from the gradients (20). After analysis of the first two components of these embeddings, the number of clusters was chosen to ensure a sound separation of the two-dimensional points. The gradients are noisy and were for visualisation purposes, therefore, smoothed using a Savitzky-Golay filter (with window length 21 and polynomial order 2) (21).

### Statistical significance for the overlap of two sets of markers

To calculate the probability of the selected markers overlapping with those from the Hannum and Horvath epigenetic clocks, one must first calculate the probability of choosing the markers from each selection and then consider the probability that a certain number of these match. This is done by consulting the Hypergeometric distribution or a normal approximation of this since the exact hypergeometric probability is difficult to calculate (due to the large products for large factorials and the problem with representing these in computers).

*x* = number of markers in common between two groups.

*n* = number of markers in group 1.

*D* = number of markers in group 2.

*N* = total number of markers The normal approximation is used when:

$$\begin{equation*} p\pm 2 \cdot \sqrt\frac{p \cdot q}{n} > 0 \, {\rm and} \, n \cdot 10 < N, \end{equation*}$$

where *p* = *D*/*N* and *q* = 1 – *p*.

The normal approximation is:

$$\begin{equation*} Z=abs\left(\frac{x-0.5-n\cdot p}{\sqrt{npq}}\right) \end{equation*}$$

Probability = *P Z* , where *Z* is a standard normal variation from *N*(0,1).

$$\begin{equation*} P{Z} = 1-\frac{erff(\frac{Z}{\sqrt{2}}+1)}{2}, \end{equation*}$$

where erff is the error function, see http://nemates.org/MA/progs/overlap_stats.html for code and calculations.

### Comparing age predictions from linear, absolute value and fold change selection

To ensure the presented absolute value and fold change selections result in more informative markers, we build machine learning models in the form of random forest regression with scikit-learn (22) using the selected markers. We compare the accuracy from our models with that of predictions using both the Hannum (5) and Horvath (3) models for blood and the Horvath model for the cerebellum and frontal cortex. We use a 5-fold cross-validation (CV) to reduce training bias and display the strength of the selected markers. We perform training on 80% of the data and validate the remaining 20% using the default parameters in scikit-learn.

### Gene Ontology enrichment

To analyze the biological importance of the selected markers, Gene Ontology (GO) enrichment was performed using PANTHER, version 14 (23). The enrichment was performed for each cluster in each tissue for the best performing selection (FC, absolute value or overlap) to analyze potential differences and similarities between markers with different age relationships.

## RESULTS

### Marker selection

For the markers that are statistically significant on false discovery rate (FDR) 0.05, there are 120 and 540 out of 432 924 methylation markers for blood, which running medians display a fold change (FC) higher than two and which have beta value changes of 0.2 for the FC and absolute value selection respectively. There are 23 overlapping markers identified between these selections. Further, 514 out of 27 476 markers from the cortex hava FC higher than two, 190 are identified by the absolute value selection, and 55 of these markers are overlapping. For the cerebellum, 152 markers are found by FC, 240 for the absolute value selection, and 55 of these markers are found by both methods.

### Blood markers and ageing

**Figure 3.**
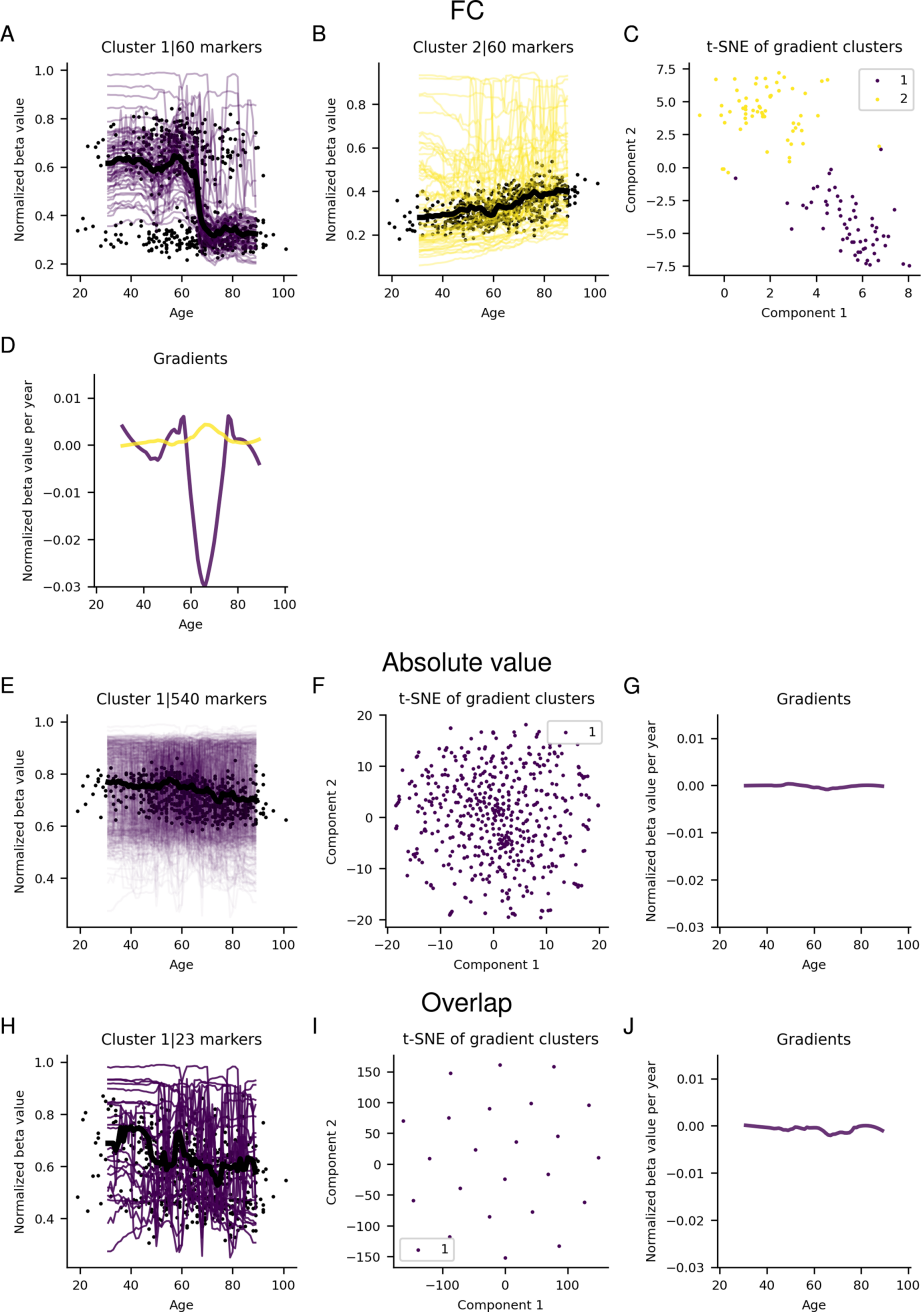
Significant running medians for marker clusters from blood for the FC (**A** and **B**) and absolute value (**E**) selections and the overlap (H) between these. The black points represent the median value for each sample, and the black lines the running median for each cluster. The beta values have been normalized with the highest beta value for each marker. (**C**), (**F**) and (**I**) visualize the t-SNE cluster embeddings on two components for the FC, absolute value and overlap selections, respectively. (**D**), (**G**) and (**J**) Gradients of running medians against age, smoothed medians using a Savitzky-Golay filter (21) with window length 21 and polynomial order 2 for the FC, absolute value and overlap selections, respectively.

For the FC selection, one of the clusters found in blood displays a negative correlation (cluster 1, 56 markers) and the other positive (cluster 2, 47 markers) correlation with age (Figure 3). These clusters show clear separation from each other and contain only a few outliers. For the positive relationship in blood, the methylation rate is steady throughout ageing. The negative relationship in the blood is a more extreme inverse of the positive, steady states before and after a large drop around age 60 are observed. The beta values for both clusters are low (0.2–0.1), with outliers of higher values (Supplementary Figure S2A and B).

Only one cluster is visible for the absolute value selection and, therefore, also in the overlap selection, Figure 3E. This cluster has 108 markers in the absolute value selection and 23 in the overlap and negatively correlates with age (Figure 3E and H). This decrease is moderate, and the cluster points for the absolute value selection are collected, comparable to that of cluster 2 in the FC selection. The gradients are small and show a slow decrease by age.

### Frontal cortex markers and ageing

**Figure 4.**
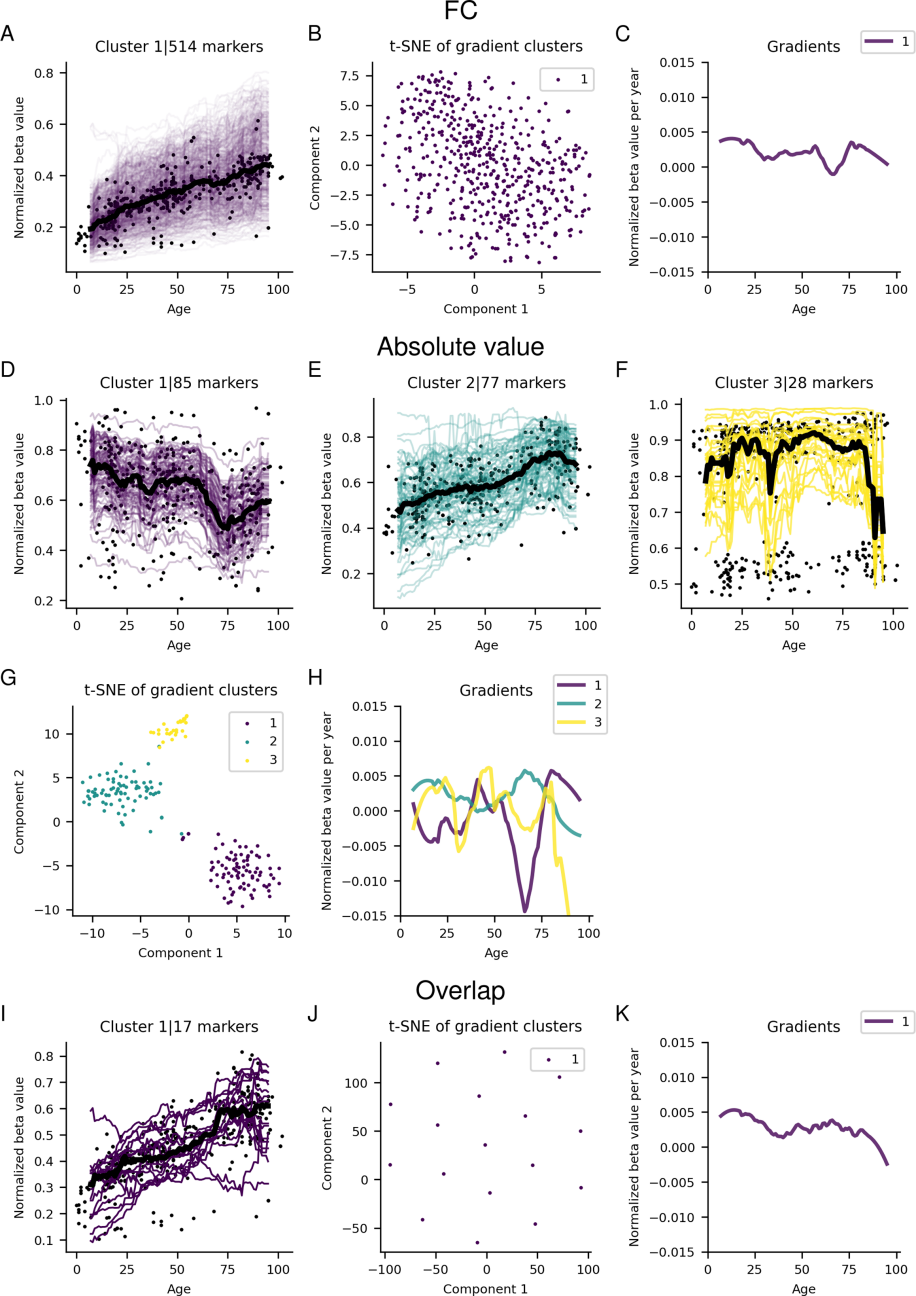
Significant running medians for marker clusters from the frontal cortex for the FC (**A**) and absolute value (**D–F**) selections and the overlap (**I**) between these. The black points represent the median value for each sample, and the black lines the running median for each cluster. The beta values have been normalized with the highest beta value for each marker. (**B**), (**G**) and (**K**) show the visualization of the t-SNE cluster embeddings on two components for the FC, absolute value and overlap selections, respectively. (D), (G) and (**J**) Gradients of running medians against age, smoothed medians using a Savitzky-Golay filter (21) with window length 21 and polynomial order 2 for the FC, absolute value and overlap selections, respectively.

For the FC selection in the frontal cortex (Figure 4A–C), only one central cluster with 514 markers was found. This cluster only displays significantly positive relationships with ageing. The gradients for this cluster are very stable throughout all ages (8–96 years). At age 8, the gradients are positive, with a median change of about 0.4% of the maximal observed beta value per year. As ageing proceeds, the gradients decline towards zero. The number of points rapidly declines, and the spread increases in this area. The beta values range from close to 0 to 0.1–0.2 (Supplementary Figure S2C).

There are three different clusters with 85, 77 and 28 markers for the absolute value selection (Figure 4D–F). Cluster 2 resembles the FC selection, while cluster 1 instead shows an initial slow decrease, followed by a rapid decrease in beta value between ages 50–75. Cluster 3 arises due to a large spread in the points making up the running median, although there is very little overall change in the running median throughout all ages. The gradients are noisy, but confirm the overall relationships as largely positive or negative with a diminishing derivative (Figure 4G). The overlap selection only has one cluster (like the FC selection) with 12 markers, showing an increasing relationship with age.

### Cerebellum markers and ageing

**Figure 5.**
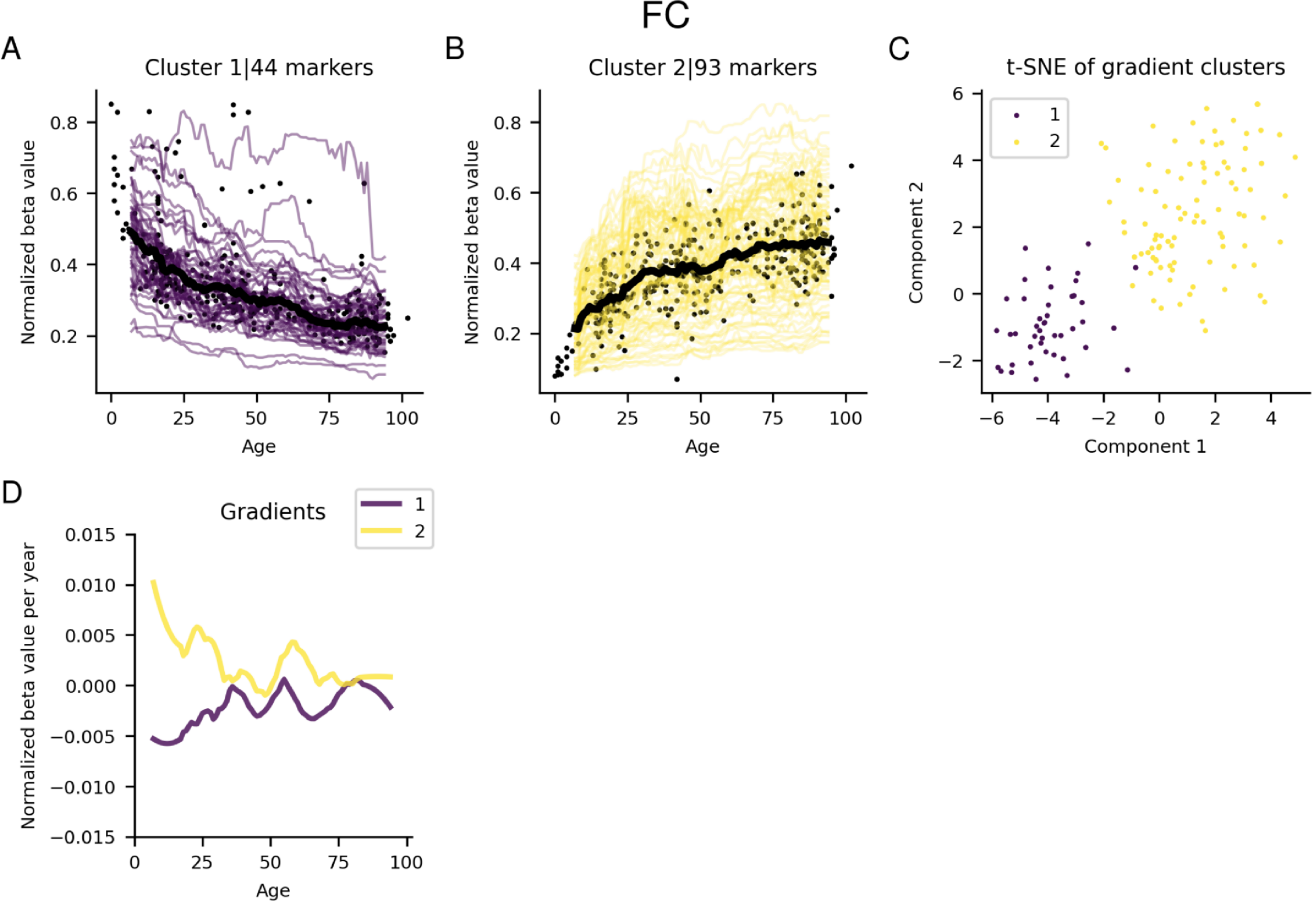
(**A–D**) Running medians for significant marker clusters from the cerebellum. The black points represent the median value for each sample, and the black lines the running median for each cluster. The beta values have been normalized with the highest beta value for each marker. (**E**) Visualization of the t-SNE cluster embeddings on two components. (**F**) Gradients of running medians against age, smoothed medians using a Savitzky-Golay filter (21) with window length 21 and polynomial order 2.

Two distinct clusters were found for the FC selection in the cerebellum (Figure 5). Cluster 1 contains 44 markers and displays a negative correlation with ageing, having increasing gradients to the age of 30, where the gradients flatten out and remain steady. Most beta values in cluster 1 range from 0.1 to 0.6 (Supplementary Figure S2D). Cluster 2 has 93 markers and shows an accelerating methylation up to age 30 and flattening out towards older ages (beta values of 0.1–0.6, Supplementary Figure S2E).

**Figure 6.**
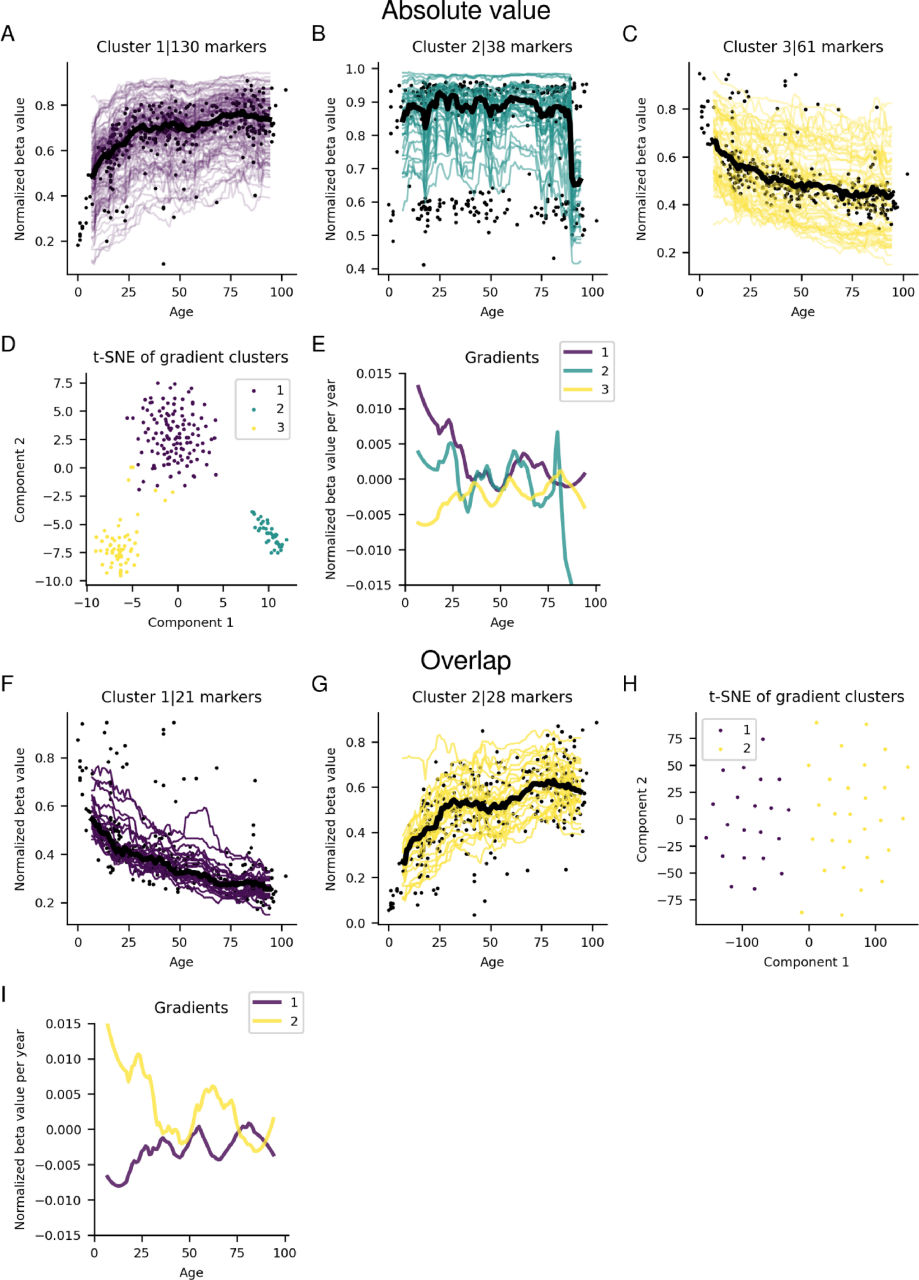
Significant running medians for marker clusters from the cerebellum for the absolute value (**A–C**) selections and the overlap (**F, G**) selections. The black points represent the median value for each sample, and the black lines the running median for each cluster. The beta values have been normalized with the highest beta value for each marker. Furthermore, (**E**) and (**H**) show the visualization of the t-SNE cluster embeddings on two components for the absolute value and overlap selections, respectively. (**D**) and (**H**) Gradients of running medians against age, smoothed medians using a Savitzky-Golay filter (21) with window length 21 and polynomial order 2 for the absolute value and overlap selections, respectively.

Using absolute value selection, three different clusters with 130, 38 and 61 markers are identified (Figure 6A–C). Clusters 1 and 3 resemble clusters 2 and 1 from the FC selection, respectively. These clusters are also present in the overlap selection (21 and 28 markers respectively, Figure 6F and G). These gradients, as a result, follow those of clusters 2 and 1 in the FC selection. Cluster 2 in the absolute value selection has 38 markers, as in the frontal cortex, this cluster arises due to a large spread in the points making up the running median, although there is very little overall change overall.

### Comparing marker selections from direct age correlations, epigenetic clocks and running medians

For the FC selection, out of the 103 significant methylation markers for blood, 514 for the frontal cortex and 137 for the cerebellum 99, 510 and 134 markers were significant on FDR 0.05 when analyzing the Pearson correlation coefficient between age and marker values.

**Figure 7.**
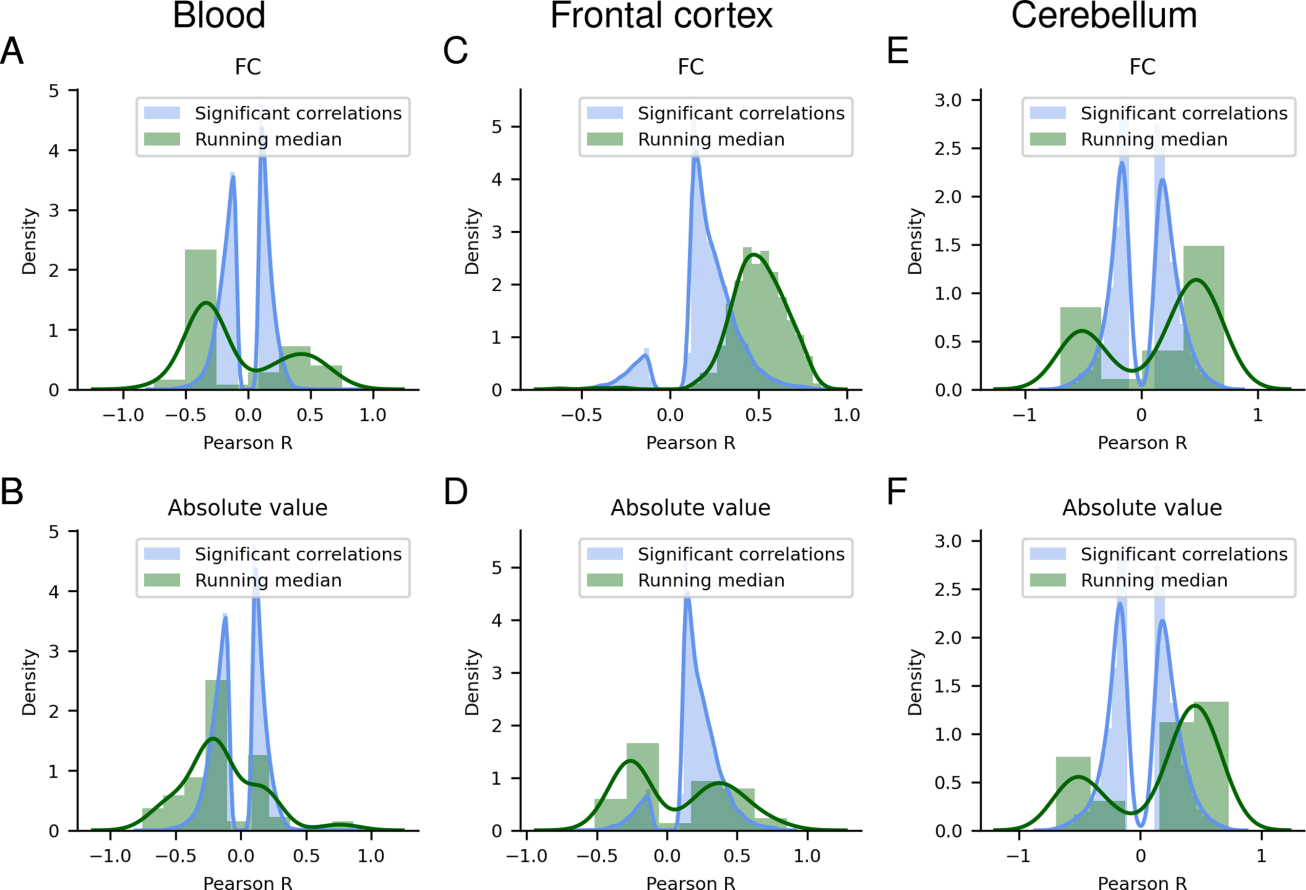
The distribution of the Pearson correlation coefficients from the markers with significant age correlations and those selected from the running medians. Blood (**A, B**), frontal cortex (**C, D**) and cerebellum (**E**, **F**), for the FC and absolute value selections respectively.

For the absolute value selection, the corresponding numbers are 84 out of 108, 153 out of 190 and 185 out of 229 markers for blood, the frontal cortex and the cerebellum, respectively. The correlation analysis deemed 170206 (44%), 10235 (39%) and 5744 (22%) unique markers significant on FDR 0.05 for blood, frontal cortex and cerebellum, respectively. When selecting markers with significant age correlations (FDR < 0.05), one obtains a bimodal distribution of Pearson correlations (Figure 7). Other distributions are obtained from both the FC and absolute value running median selections, showing that even poorly correlated markers can portray significant methylation changes. However, the frontal cortex selection shows almost only positively correlating markers for the FC selection.

**Figure 8.**
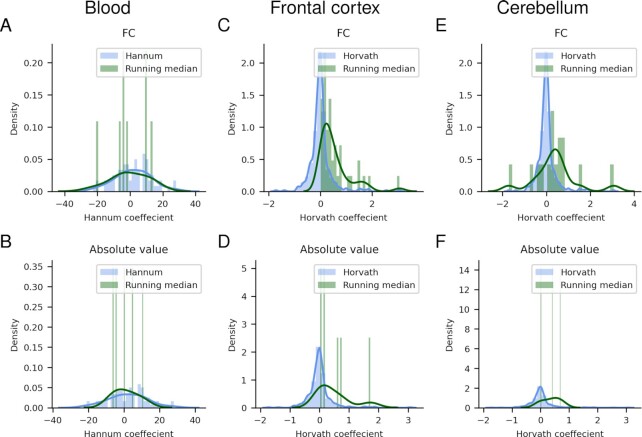
The coefficients for the linear marker combination in the Hannum (71 markers) and Horvath (353 markers) clocks and the overlapping markers selected from the running medians in blood (**A**, **B**), frontal cortex (**C**, **D**) and cerebellum (**E**, **F**) for the FC and absolute value selections, respectively.

For the FC selection, of the 103 significant blood markers, eight overlap with the 71 markers used in the Hannum epigenetic clock (5), all belonging to cluster 2 (Figure 3). Of the 514 and 137 significant markers in the frontal cortex and cerebellum, 7 and 15 overlap with the 353 markers used in the Horvath epigenetic clock (3), respectively. All seven in the frontal cortex belong to the only cluster (Figure 4), while of the 15 in the cerebellum 11 belong to cluster 2 and 4 to cluster 1 (Figure 5). For the absolute value selection, only 2 out of 108 significant blood markers overlap with the Hannum markers and belong to cluster 1 (Figure 3). Seven out of 190 markers overlap with the Horvath markers in the frontal cortex, and 2 out of 229 in cerebellum (Figures 4 and 6). The overlapping markers have positive or negative coefficients for the cerebellum and blood, while the frontal cortex has only positive correlations (Figure 8). The probability of selecting the significant markers randomly and that the observed overlap with the epigenetic clock markers for the FC selection would be found is *P* < 1.870 × 10^−19^, *P* < 0.469 and *P* < 4.987 × 10^−10^ for blood, frontal cortex and cerebellum respectively (see Materials and Methods). For the absolute value selection, these numbers are *P* < 0.019, *P* < 0.014 and *P* < 0.409 for blood, frontal cortex and cerebellum respectively. The only significant overlap is thus from the FC selection for blood and cerebellum. None of the overlaps with the absolute value selection are significant, suggesting that the FC selection has a higher correspondence with the epigenetic clock markers than the absolute value selection.

**Figure 9.**
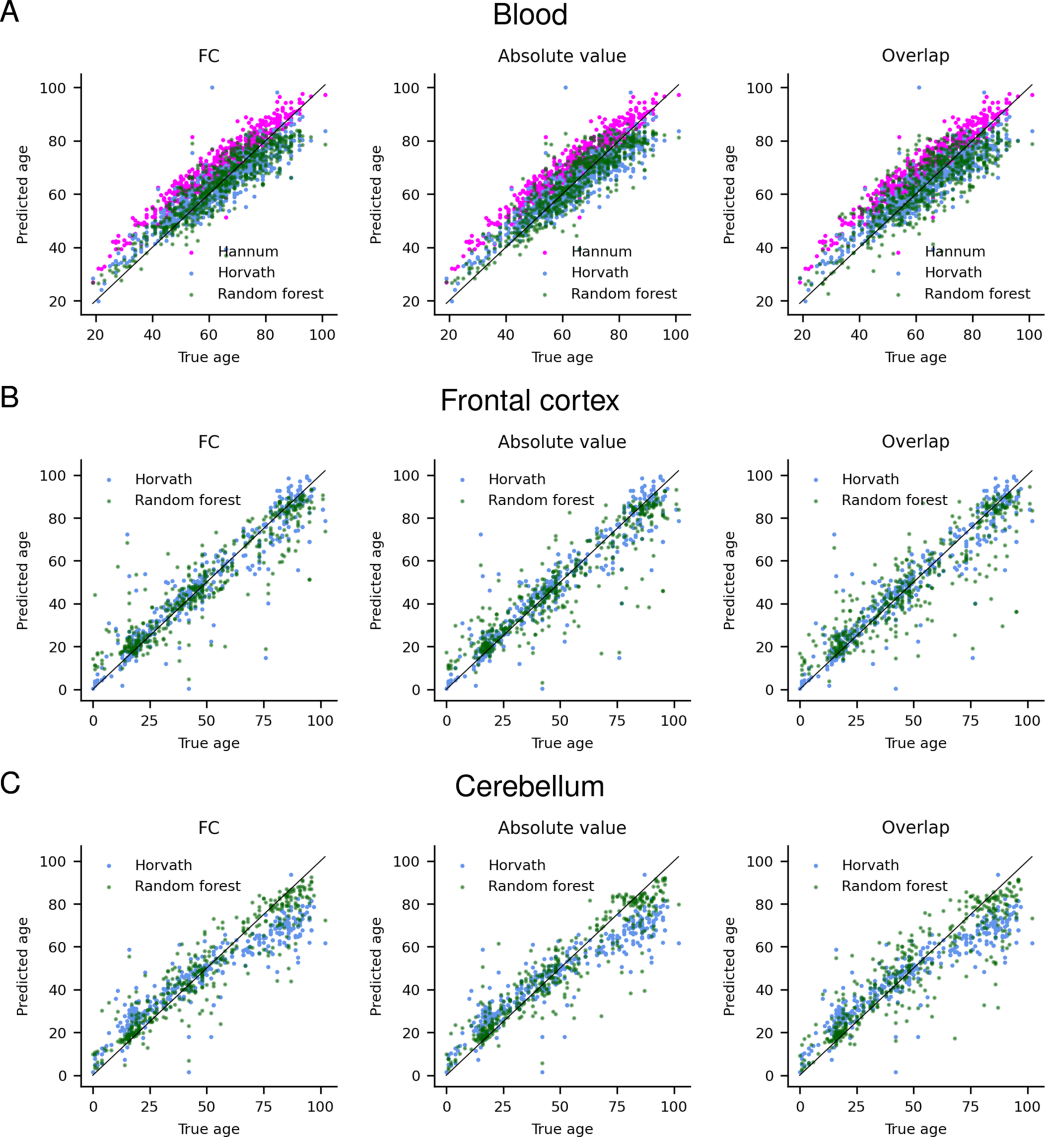
Results from fitting a random forest regressor to the blood (**A**), frontal cortex (**B**) and cerebellum (**C**) markers and comparing with the Hannum (blood) and Horvath (blood, frontal cortex and cerebellum) results for the FC, absolute value and overlap selections respectively. One can see that the results are substantially improved using the running median selection method for the cerebellum but not for the frontal cortex or blood.

### Comparing the prediction accuracy between epigenetic clocks and running median selections

The results from fitting a random forest regressor to the running median selections display a decrease in error only for the cerebellum (Table 1, Figure 9). The errors are consistently lower for the FC selection across tissues, and higher for the overlap selection. Compared to the Horvath clock, a reduction in error is obtained using the FC selection for the cerebellum (6.66 versus 9.48 years), but a slightly higher error for the frontal cortex (6.79 versus 5.35 years). For blood, the error is 5.52 years for the Hannum clock and 4.85 for the Horvath clock versus 4.99 years for the running median selection, although the Hannum clock has been trained on the same dataset (5). The Pearson correlation coefficients are lower for the random forest models in blood and the frontal cortex but higher in cerebellum. The previously reported underpredictions for the cerebellum (8) manifest here as well.

**Table 1.** Average errors and Pearson correlations for the Hannum, Horvath and random forest models fitted to each tissue type and for the FC, absolute value and overlap selections, respectively

Model	Error	PCC
Blood Hannum	5.52	0.95
Blood Horvath	4.85	0.91
Blood FC	4.99 ± 0.23	0.91 ± 0.01
Blood absolute value	5.06 ± 0.49	0.90 ± 0.01
Blood overlap	6.55 ± 0.22	0.83 ± 0.01
Frontal cortex Horvath	5.35	0.95
Frontal cortex FC	6.79 ± 0.61	0.92 ± 0.02
Frontal cortex absolute value	7.04 ± 1.06	0.92 ± 0.03
Frontal cortex overlap	7.68 ± 0.89	0.91 ± 0.02
Cerebellum Horvath	9.48	0.93
Cerebellum FC	6.66 ± 0.76	0.94 ± 0.01
Cerebellum absolute value	6.72 ± 0.57	0.94 ± 0.01
Cerebellum overlap	8.14 ± 0.73	0.91 ± 0.02

### Genes regulated by significant methylation markers and GO enrichment

Since several markers may regulate the same gene, the markers were grouped by gene for the best performing FC selection and further analysed. The relationship of all genes with at least two significant methylation markers was assessed to investigate potential differences in methylation change for these markers. There are 88, 438 and 127 unique genes for blood, frontal cortex and cerebellum, respectively. In blood, 3 out of 88 genes are regulated by two significant markers, 32 out of 438 for the frontal cortex and 5 out of 140 for the cerebellum. The 3, 32 and 5 genes are regulated by 7, 65 and 10 markers, respectively. All of these show positive relationships with age (Supplementary Figures S3–S5), belonging to clusters 2, 1 and 2 for blood, frontal cortex and cerebellum, respectively.

GO enrichment of the unique genes suggests what processes the markers regulate (Supplementary Figures S6 and S7). The two marker clusters for blood are mostly related to metabolic and cellular processes and biological regulation with similar frequencies. This is valid also for the Hannum selection, although cluster 1 and the Hannum selection show relationships with more divergent processes than cluster 2. The frontal cortex enrichment (only one cluster) shows various processes, substantially overlapping with the Horvath marker GO terms. The two different cerebellum clusters show a wide variety of GO terms, although metabolic processes, cellular processes and localization are important in all. Both clusters overlap with the Horvath terms. All clusters show essential differences, supporting the validity of their division.

## DISCUSSION

An issue with investigating marker correlation with age is not evaluating the fold change (FC), only the correlation itself. A very high number of markers are significant on FDR 0.05 from the Pearson correlation analysis. However, a marker that changes only 1% in total but does so with 0.01% a year from year 0-100 will have a Pearson correlation coefficient of 1.0 with age. When evaluating the FC, larger relative methylome changes are assessed, capturing changes that likely have a higher impact on gene expression. An issue with both FC and correlation selection is that the absolute value change might be minimal. To address this issue, we analyzed the outcome of both absolute value selection and the overlap between absolute value and FC selections, finding that FC selections are the most robust and produce the lowest errors when applied to biological age prediction.

The number of individuals for each age is not uniform, which is usually the case in clinical studies (see Figure 2). We solve this problem by selecting 10% of the closest samples in age for each year, thus creating equal sample representations for each age. This selection may, on the other hand, result in the methylation values from under-represented age groups not being considered appropriately. The alternative is to represent each age with very few samples, which we find worse. Further, we ensure all significant changes throughout ageing are assessed by analysing the running median across all ages. A *t*-test of the significance between the samples is used to construct the median points related to the FC, and absolute value selections ensure statistical soundness.

To investigate all possible significant relationships between ageing and methylation change, *k*-means clustering was performed on the normalized gradients for all selections. The number of clusters was evaluated through t-SNE, ensuring sufficient separation between gradient clusters. No cluster in any tissue displays methylation-age relationships equal to another. However, there are similarities, e.g. cluster two in blood, one in frontal cortex and three in cerebellum are all very similar for the FC selection. This suggests age-related methylation is highly tissue-specific, as suggested from studies of tissue-specific age predictors (7).

In the cerebellum, all methylation-age relationships have a tendency for saturation as ageing proceeds, explaining why there is a consistent underprediction of the age in older samples for the epigenetic clocks that use linear combinations of methylation change (8). This tendency should not be due to the underrepresentation of older samples, as the equal age representations and control for edge effects (see Materials and Methods) prevent such issues. However, we did not perform covariate adjustments for e.g. BMI due to lack of data, meaning that some inter-individual variability may not be considered. Regardless, no relationship is strictly linear which is why such adjustments may not be meaningful, although the one found in the frontal cortex is quite close. However, analysis of the gradients provides a clearer picture of how the medians change, which is difficult to capture when analyzing only the direct relationships.

All tissues contain clusters that have linear regimes, although the cerebellum clusters display largely non-linear relationships with ageing (Figure 5). The linear regimes explain why the epigenetic clocks and age adjustments in previous studies (3,5,9–13) work, although predominantly markers with non-significant changes are included.

Blood is the tissue with the most protrusive methylation relationships. Why the methylation levels change rapidly at age 60 is unclear. An explanation may be a downregulation of immune responses at older ages (24), as DNA from blood is mostly from leukocytes (25), although this requires further studies to draw any conclusions. The running medians constituting the blood clusters have substantial spread as well. This creates noise for the total cluster medians (black lines in Figures 3–5), impacting the observed relationships. The Savitzky-Golay filter applied to the gradients should be able to counteract such noise, potentially capturing significant signals.

The frontal cortex displays only hypermethylation for the FC selection, while blood and cerebellum display both hypo- and hypermethylation. Although, in the absolute value selection, the frontal cortex cluster one exhibits hypomethylation. Since hypomethylation is related to increased gene expression (26,27), the continuous hypermethylation observed, in the frontal cortex by the FC selection, suggests gene expression is continuously downregulated. This corresponds well with previous findings in the prefrontal cortex, where DNA methylation changes have been found to be fast during the prenatal period, and later slow down and do so continuously with ageing (28).

The beta values were normalised to enable comparison of relative change in methylation and thus obtain comparable gradients. In Supplementary Figure S2, unnormalised relationships are displayed for the FC selection. The beta values of the selected markers for blood and the frontal cortex are very similar for the majority of the running medians but low, only amounting to maximal 20% methylation. The beta values for the selected markers in the cerebellum, on the other hand, are both larger and show greater variability, most ranging from 0.1 to 0.5. This suggests that the gene expression in the cerebellum may be more drastically altered during ageing, compared with the smaller changes observed in the other tissues.

When comparing the significant markers for the FC selection with those of the Hannum (only blood) (5) and Horvath (multi-tissue) (3) epigenetic clocks, there is significant overlap only from the FC selection for blood and cerebellum. Despite the significant overlap, few markers overlap. This suggests that most of the significant changes are missed by linear analysis. The argument for selecting markers that correlate highly with age is thus still without support, something highlighted in the non-linear findings here. This holds for the absolute value selection as well since the markers do not display significant overlaps.

The FC selection random forest prediction errors are only better in the cerebellum compared to the Horvath and Hannum clocks. This is likely due to the largely non-linear relationships observed. However, the Horvath clock was created using fewer markers (21 639 versus 26 467). It should be noted that the markers selected here were also not chosen for the objective of predicting age, which is the case in the epigenetic clocks. This suggests that selecting markers based on fold change enables capturing biological age relationships, while linear selection does not manage when the age relationships happen to be essentially non-linear.

The GO enrichment for the FC selected markers shows sound annotations for all tissues and epigenetic clock comparisons. Interestingly, different clusters display GO terms related to different biological processes within the same tissue. The overlap with the epigenetic clocks is largest for the clusters with more linear relationships. Without evaluating the effect size of the methylation change, it is difficult to know if a marker will have much impact on gene expression or development.

## CONCLUSIONS

Here, we provide a new way to investigate all possible methylation-age relationships and group them according to their relative change rates. Across all tissues, the fold change (FC) selection consistently outperforms the absolute value and overlap selections for biological age prediction. This suggests that the FC selection is a more robust selection method of these three. The relationship between significant DNA methylation changes and ageing varies between tissues and life periods. The variability of the relationship between methylation and age, especially the saturation observed at older ages, might provide important insights. The regions of semi-linearity explain why epigenetic clocks and age adjustments that assume linear relationships produce successful results. The saturation explains why ageing is underpredicted in older samples, especially in the cerebellum. When analyzing the relationship between methylation levels and age, one has to evaluate not only linear relationships between DNA methylation and age but also non-linear relationships. We show that the FC selection outperforms the epigenetic clocks in predicting age in the cerebellum. This is true even though the objective of age prediction was not sought after, and no parameter optimization was performed for the models built with these markers. In blood and the frontal cortex the most common epigenetic clocks are more successful however.

## DECLARATIONS

### Ethics approval and consent to participate

The data is from previous studies, available from ArrayExpress. We refer to the accession numbers E-GEOD-40279, E-GEOD-36194 and E-GEOD-15745.

## DATA AVAILABILITY

The datasets used, E-GEOD-40279 (https://www.ebi.ac.uk/arrayexpress/experiments/E-GEOD-40279/), E-GEOD-36194 (https://www.ebi.ac.uk/arrayexpress/experiments/E-GEOD-36194/) and E-GEOD-15745 (https://www.ebi.ac.uk/arrayexpress/experiments/E-GEOD-15745/), are available at the ArrayExpress database. The code is freely available under the GPLv3 license: https://github.com/patrickbryant1/ageing_of_blood_and_brain.

## Supplementary Material

lqac001_Supplemental_File
